# Supplementary material for: Evidence-based practice by physiotherapists in UAE: Investigating behavior, attitudes, awareness, knowledge and barriers
Source: PLoS One. 2021 Jun 18;16(6):e0253215. doi: 10.1371/journal.pone.0253215 (PMC8213175; doi:10.1371/journal.pone.0253215)
Supplement: S1 Appendix — (PDF) [file pone.0253215.s001.pdf]

S1 APPENDIX. Questionnaire

Investigating Behavior, Attitudes, Awareness, Knowledge and Barriers to the Implementation of Evidence-Based Practice by Physiotherapists in UAE: A Cross Sectional Study

This survey consists of <u>15 QUESTIONS</u> and will take <u>LESS THAN 8 MINUTES</u> to complete.

<u>This survey is not suitable for BSc students.</u>

The aim of this survey is to identify physiotherapists' awareness and knowledge with regard to the implementation of evidence-based practice (EBP) in UAE. Physiotherapists of all nationalities in UAE are welcomed to participate irrespective of whether you are working in a clinical or an academic setting.

The data will be stored by the researcher and will form part of a written report used for research purposes. All participants will be anonymized.

<u>Your responses will help us to improve the quality of physiotherapy services in UAE.</u>

هذا الاستبيان يشمل 15سؤال ويمكن تعبئته خلال فترة زمنية لا تتعدى 8 دقائق.

هذا الاستبيان ليس مناسبا لطلاب مرحلة البكالوريوس.

الهدف من هذا الاستبيان هو تحديد مدى معرفة ووعي ممارسي العلاج الطبيعي تجاه الممارسة التطبيقية المبنية على الأدلة. جميع الجنسيات هم موضع ترحيب لملء هذا الاستبيان فقط إذا كنت تعمل في الامارات العربية المتحدة سواء في المجال الاكلينيكي أو الأكاديمي.

سيتم تخزين البيانات الكترونيا من قبل الباحث، ستشكل البيانات جزء من بحث منشور بإذن الله لأغراض بحثية وتطويريه، ولكن ستبقى معلوماتك الشخصية مجهولة المصدر.

مشاركتك تهمنا وبعد توفيق الله ستكون لها دور فعال للمساعدة في تطوير مهنة العلاج الطبيعي في الامارات العربية المتحدة.

- **Question 1 of 15: What is your gender?**

Male

Female

- **Question 2 of 15: What is your age?**

20 - 25

26 - 30

31 - 35

36 - 40

41 or more

- **Question 3 of 15: What is your nationality?**

- **Question 4 of 15: What is your highest level of education?**

Diploma

Bachelor's degree

DPT degree

Master's degree

PhD degree

Bachelor's degree + DPT

Master's degree + DPT

PhD degree + DPT

- **Question 5 of 15: From which university did you obtain your highest degree?**

- **Question 6 of 15: Which of the following best describes your main work setting?**

Ministry of Health and Prevention (MOHAP)

Dubai Health Authority (DHA)

Abu Dhabi Healthcare Company (SEHA)

Private health sector

University/ Collage (Academic institution)

Other (please specify)

- **Question 7 of 15: What is your job title for your main work setting?**

Junior Physiotherapist

Senior Physiotherapist

Specialist Physiotherapist

Consultant Physiotherapist

Tutor

Lecturer

Assistant Professor

Associate Professor

Professor

- **Question 8 of 15: Years of experience**

1-5 years

6-10 years

11-15 years

16-20 years

21-25 years

>25 years

- **Question 9 of 15: Please rate your responses to the following statements:**

	**Strongly disagree.**	**Disagree**	**Neutral**	**Agree**	**Strongly agree**
Understanding of research methods and research designs is important in physiotherapy practice.					
Research theory and methodology should be included in the physiotherapy curriculum.					
Physiotherapists need to read relevant articles regularly to update their knowledge.					
Physiotherapists should apply treatments that are supported by					

evidence.					

- **Question 10 of 15: Please rate your use of the following sources when you make a clinical decision?**

	**Always**	**Often**	**Sometimes**	**Rarely**	**Never**
My personal experience					
My colleagues' opinions					
My supervisor's or expert opinions					
Internet					
Books					
Research reviews and articles					

Please Notice:

EBP = Evidence-based practice

- **Question 11 of 15: How familiar are you with the following terminologies and phrases?**

	**Never heard it.**	**Have heard it but don't understand.**	**Understand a little**	**Understand very well**	**Understand completely and could explain to others**
**EBP as a term**					

**EBP cycle/steps**					
**Quality of evidence**					
**Systematic review**					
**Randomized controlled trial**					
**PICO**					
**Critical Appraisal**					
**Forest plot**					
**Relative risk**					
**Likelihood ratio**					
**Confidence interval**					
**Effect size**					
**Risk of bias**					
**Healthcare databases such as MEDLINE, PEDro, etc.**					

- **Question 12 of 15: Please select your answer to the following statements:**

	**Agree**	**Disagree**	**Unsure**
EBP is a process of systematic investigation to generate knowledge and test theories.			
The main aim of EBP is to identify the causes of research problems and how to solve them.			
Physiotherapy interventions are mostly.			

supported by EBP.			
Patient's values and preferences are not one of the main requirements of EBP.			
EBP does not take into consideration the clinical experience of the physiotherapist.			
EBP requires a short period of time to search for, evaluate and integrate evidence into practice.			

- **Question 13 of 15: Have you formally undertaken any training in EBP?**

Yes

No

Question 14 of 15: If YES, what type of training course have you been involved in? (If NOT, skip this question)

EBP course as part of University education 'BSc, MSc, PhD' (more than 20 hours)

Comprehensive course (11 to 20 hours)

Short course (3 to 10 hours)

One lecture (1 to 2 hours)

Other (please specify)

- **Question 15 of 15: Please rank your response with regard to possible barriers to research and EBP knowledge, and their application where 10 is the most important choice as far as you are concerned, and 1 is the least important.**

	1 Least Important	2	3	4	5	6	7	8	9	10 Most important
Lack of research knowledge and skills										
Lack of support and encouragement										
Insufficient teaching in previous education										
Lack of time										
Lack of interest										
Lack of funding and resources such as access to databases and journals										

Thank you for taking the time to complete our survey

If you require any further information, feel free to contact:

Hamda Khalifa AlKetbi

Email: U18105988@sharjah.ac.ae

Mobile phone: 052-8800018

Please feel free to leave a message or a comment:
